# Complete mitochondrial genomes of two insular races of *Pazala* swordtails from Taiwan, China (Lepidoptera: Papilionidae: *Graphium*)

**DOI:** 10.1080/23802359.2021.1915719

**Published:** 2021-04-26

**Authors:** Shao-Ji Hu, Xin Zhang, Kuang Duan

**Affiliations:** a Institute of International Rivers and Eco-security, Yunnan University, Kunming, China; b Yunnan Key Laboratory of International Rivers and Transboundary Eco-security, Yunnan University, Kunming, China; c Kunming Youning Biotech Co., Ltd, Kunming, China; d School of Agriculture, Yunnan University, Kunming, China

**Keywords:** Sino-Himalaya, insular endemic subspecies, protein coding genes, transfer RNA, ribosomal RNA

## Abstract

The mitogenomes of two insular subspecies of *Pazala*, *G.* (*P.*) *eurous asakurae* and *G.* (*P.*) *mullah chungianus* from Taiwan Island, are reported. Both mitogenomes are circular, 15,228 bp and 15,240 bp in length respectively, and consist of 37 genes, including 13 PCGs, 22 tRNAs, and two rRNAs. The Bayesian phylogenetic tree containing the focal taxa and 31 other Papilioninae members clustered them with *G.* (*P.*) *mullah* (Alphéraky, 1897) and then *G.* (*P.*) *parus* (Nicéville, 1900) inside tribe Leptocircini, which agrees with their taxonomic positions. The findings of this study would benefit future understanding of phylogeography and conservation of subgenus *Pazala*.

Taiwan Island is the easternmost point in the distribution range of the swordtail butterflies of subgenus *Pazala* Moore, 1888 (Lepidoptera: Papilionidae), represented by two endemic subspecies, *Graphium* (*Pazala*) *eurous asakurae* (Matsumura, 1908) and *G.* (*P.*) *mullah chungianus* (Murayama, 1961) (Racheli and Cotton 2009; Hsu et al. 2018). Although the endemicity of some *Pazala* taxa has been partly discussed in recent taxonomic works (Hu et al. 2018, 2019; Zhang et al. 2020), the phylogeographic history of this intriguing Sino-Himalayan swallowtail group is still poorly understood. The mitogenome contains very useful phylogenetic information for evolution, and has become a feasible tool in phylogeography. The complete mitogenomes of the two insular endemic subspecies of *Pazala* reported herein would facilitate future research related to in-depth butterfly conservation (Wang et al. 2020).

The samples used in this study were collected from Taiwan Island. *G.* (*P.*) *eurous asakurae* came from Taroko, Hualien (24.202133°N, 121.454329°E), and *G.* (*P.*) *mullah chungianus* was collected at Fushan, Yilan (24.749637°N, 121.639251°E). The specimens were deposited in the zoological museum (insect collection) of Yunnan University, Kunming, China (specimen numbers: YNU-LEP-PAP-2019028 and YNU-LEP-PAP-2019102, contact person: Shao-Ji Hu). Genomic DNA was extracted from the thoracic muscle tissue of the adult using phenol-chloroform and isopropanol protocol (Hu et al. 2013) and sequenced on an ABI 3730xl automatic sequencer (Applied Biosystems, CA, USA). Resultant gene fragments were assembled using DNAStar (https://www.dnastar.com/) with the previously reported mitogenomes of *G.* (*P.*) *mullah* (Alphéraky, 1897) (KJ472924) (Chen et al. 2014) and *G.* (*P.*) *parus* (Nicéville, 1900) (MT198821) (Duan et al. 2020) as references. Protein-coding genes (PCGs), transfer RNA genes (tRNAs) and ribosomal RNA genes (rRNAs) were predicted using the web based MITOS (http://mitos.bioinf.uni-leipzig.de/index.py) (Bernt et al. 2013) and the Alignments | CDS feature under BLASTn of NCBI (https://blast.ncbi.nlm.nih.gov/).

The complete mitogenomes of *G.* (*P.*) *eurous asakurae* (MW549198) and *G.* (*P.*) *mullah chungianus* (MW549197) are circular, 15,228 bp and 15,240 bp in length respectively. The base composition of *G.* (*P.*) *eurous asakurae* is 39.81% for A, 40.58% for T, 7.85% for G, and 11.76% for C; while that of *G.* (*P.*) *mullah chungianus* is 40.11% for A, 40.85% for T, 7.60% for G, and 11.43% for C. Both mitogenomes contain 37 genes, including 13 PCGs, 22 tRNAs, and two rRNAs, plus a non-coding control region. The plus (+) strands of both mitogenomes encode nine PCGs (*nad2*, *cox1*, cox2, *atp8*, *atp6*, *cox3*, *nad3*, *nad6*, and *cob*), while the minus (–) strands encode four PCGs (*nad5*, *nad4*, *nad4l*, and *nad1*). The gene arrangement and character of both genomes agree with recently reported ditrysian Lepidoptera mitogenomes (Cao et al. 2012; Wang et al. 2019; Chen et al. 2020).

To validate the two mitogenomes, a Bayesian phylogenetic tree was reconstructed using PhyloSuite 1.2.2 (Zhang et al. 2020) using the 37 genes (13 PCGs, 22 tRNAs, and two rRNAs) with the GTR + I + G model for 1,000,000 generations. Thirty-one species of Papilioninae with available mitogenomes were used as ingroups and *Parnassius apollo* Linnaeus, 1758 (Parnassiinae; KF746065) (Chen et al. 2014) was chosen as the outgroup. The result clustered the focal subspecies with *G.* (*P.*) *mullah*, while *G.* (*P.*) *parus* is placed in the basal position of *Pazala*. All *Pazala* taxa are related to other *Graphium* species within Leptocircini, forming a monophyletic clade, supported by the maximal support values (Figure 1).

**Figure 1.**
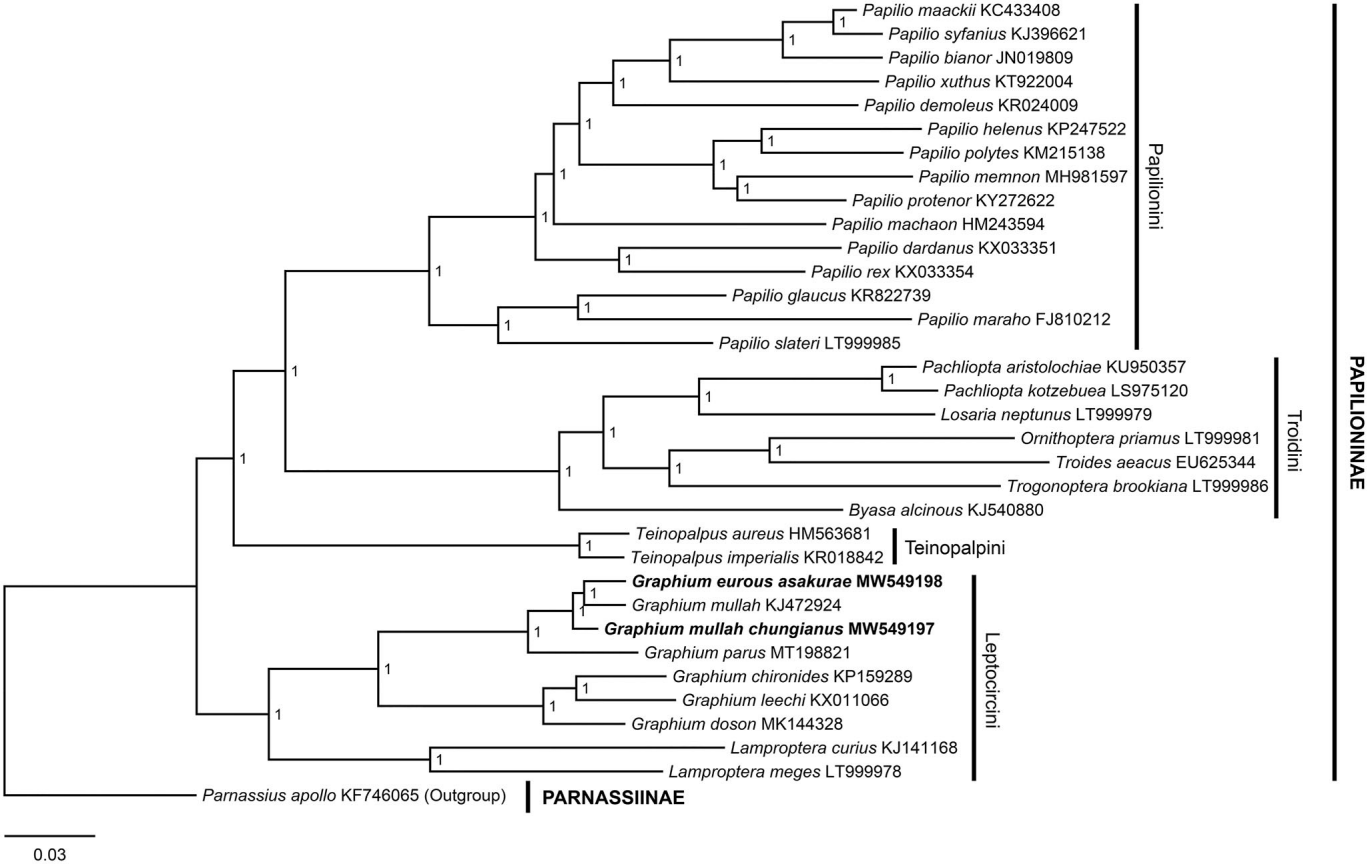
The Bayesian phylogenetic tree for *Graphium* (*Pazala*) *eurous asakurae* (Matsumura, 1908) and *G.* (*P.*) *mullah chungianus* (Murayama, 1961) (marked with bold font) and other Papilioninae taxa. Node labels represent support values.

## Data Availability

The data that support the findings of this study are openly available in the NCBI GenBank at https://www.ncbi.nlm.nih.gov/genbank/, accession numbers of all used sequences are listed in Figure 1.
